# Expression of CD147 and matrix metalloproteinase-11 in colorectal cancer and their relationship to clinicopathological features

**DOI:** 10.1186/s12967-015-0702-y

**Published:** 2015-10-27

**Authors:** Xiuyun Tian, Chunxiang Ye, Yongyong Yang, Xiaoya Guan, Bin Dong, Min Zhao, Chunyi Hao

**Affiliations:** Key Laboratory of Carcinogenesis and Translational Research, Ministry of Education/Beijing, Department of Hepato-Pancreato-Biliary Surgery, Peking University Cancer Hospital and Institute, Beijing, People’s Republic of China; Department of Gastroenterological Surgery, Peking University People’s Hospital, Beijing, People’s Republic of China; Key laboratory of Carcinogenesis and Translational Research (Ministry of Education/Beijing), Department of Biochemistry and Molecular Biology, Peking University Cancer Hospital & Institute, Beijing, People’s Republic of China

**Keywords:** Colorectal cancer, Immunohistochemistry, CD147, MMP-11, Prognosis

## Abstract

**Background:**

This study aimed to investigate the expression of CD147 and MMP-11 in human colorectal cancer (CRC) and to evaluate their clinical significance.

**Methods:**

Real-time polymerase chain reaction was used to evaluate CD147 and MMP-11 mRNA level in 56 pairs of fresh CRC samples matched with adjacent normal mucosa. The protein expression of CD147 and MMP-11 in CRC specimens and corresponding normal colorectal mucosa were evaluated by immunohistochemistry on CRC tissue microarrays. Expression and co-localization of these two proteins in human colorectal cancer tissue were also evaluated by laser scanning confocal microscopy. Furthermore, their correlations with clinicopathological factors and overall survival after surgery were evaluated.

**Results:**

Both CD147 and MMP-11 were demonstrated to be over-expressed at mRNA level (*P* < 0.001, both) and protein level (*P* < 0.001, both) in CRC tissue than paired normal mucosa. Spearman rank test showed a positive correlation between these two proteins (P = 0.025). Immunofluorescence double staining confirmed the co-localization of CD147 and MMP-11 in paraffin-embedded tissues of CRC patients. Expression of CD147 and MMP-11 were both correlated with CRC lymph node metastasis (*P* = 0.021 and *P* = 0.031, respectively), distant metastasis (*P* < 0.001 and *P* = 0.013, respectively) and TNM stage (*P* = 0.006 and *P* = 0.049, respectively). Univariate survival analysis showed that both CD147 and MMP11 expression was significantly associated with shorter survival time (*P* = 0.001 and *P* = 0.009, respectively). Additionally, in multivariate analysis, both CD147 and MMP-11 were proved to be independent prognostic factors (*P* = 0.009, 0.028, respectively).

**Conclusions:**

These results indicated that both CD147 and MMP-11 may be involved in the progression of colorectal cancer, and they are potential prognostic factors and might become new therapeutic targets for CRC patients.

## Background

Colorectal cancer is one of the most prevalent malignant tumors and a main cause of cancer related death worldwide. Its incidence has been increasing in China in recent years. Although most patients at early stage can be successfully cured with surgery, about 20–45 % of patients who underwent curative resection developed recurrence [1]. What’s more, about 15–25 % of all CRC patients have a synchronous liver metastasis, of which 80–90 % are unresectable [2, 3].Elucidation of the malignant biological behavior, especially the metastasis mechanism, is expected to bring more benefit to the CRC patients in the long run.

Matrix metalloproteinases (MMPs) are a family of zinc-dependent endopeptidases that have been extensively studied during the past years, and a variety of studies have established their crucial roles in the invasion and metastasis process of malignant tumors through degradation of the extracellular matrix (ECM) and some other mechanisms. MMP-11 (also known as stromelysin-3) is a member of the MMPs family. It was originally identified as a highly expressed protein in the stromal cells of invasive breast cancer compared to that of fibroadenoma as determined by subtractive hybridization [4]. Unlike other MMPs, MMP-11 has some unique properties. While most MMPs are secreted as inactive zymogens, MMP-11 is released as a 45 kDa active enzyme [5]. In particular, MMP-11 does not appear to degrade any classical extracellular matrix component, instead, it catalyzes the degradation of serine protease inhibitors, a 1-antitrypsin and insulin-like growth factor binding protein-1 (IGF-BP-1) [6, 7], and it may probably only play an indirect role in ECM remodeling, which is different from other MMPs. These unique properties indicate that MMP-11 might play some unique roles in malignant tumor development and progression. Previous studies have investigated its role in breast cancer [8, 9] and gastric cancer [10], while its role in colorectal cancer has rarely been investigated.

The expression and activity of MMPs are regulated under a complex mechanism, which involves a lot of signal pathways and related molecules [11], of which CD147 plays a key role. CD147, also known as extracellular matrix metalloproteinase inducer (EMMPRIN), is a member of the immunoglobulin superfamily of adhesion molecules. It plays an essential role in tumor progression and metastasis through stimulating tumor cells to secrete matrix metalloproteinases (MMPs). The relationship between CD147 and MMP-9, MMP-2 and their relevance with patients’ prognosis and clinicopathological parameters have been studied in a variety of cancers. However, the correlation between CD147 and MMP-11 expression has rarely been investigated until now.

In the present study, we investigated CD147 and MMP-11 expression in colorectal cancer. We aimed to make it clear the expression level of these two proteins in CRC tissue compared with normal mucosa, and their relationship with the clinicopathological parameters and prognosis of the CRC patients, which could provide more information for elucidating the molecular mechanism and target therapy of the disease.

## Methods

### Patients and samples

A total of 218 CRC patients who were diagnosed and underwent surgery in Peking University Cancer Hospital between 2006 and 2009 were included in this study. Fresh colorectal carcinoma specimens and paired adjacent normal mucosa from each patient were formalin-fixed, paraffin-embedded, and constructed into tissue microarrays. Postoperative follow-up has lasted at least 3 years for all of these patients. Patients who received chemotherapy or radiation therapy before surgery were excluded. Histopathological evaluation was carried out independently by two pathologists. This study was approved by the institutional review board of Peking University Cancer Hospital, and written informed consent was acquired from each patient. The characteristics of all the patients are shown in Table 1.

**Table 1:** Summary of patient characteristics (n = 218)

Clinicopathological features	No. of patients	(%) of patients
Gender		
Male	129	59.17
Female	89	40.83
Age		
<60	99	45.41
≥60	119	54.59
Tumor size (cm)		
<4	104	47.71
≥4	114	52.29
Lymphovascular invasion		
Absent	149	68.35
Present	69	31.65
Differentiation		
Well-moderate	179	82.11
Poor	39	17.89
Depth of invasion		
T1 + T2	33	15.14
T3 + T4	185	84.86
Lymph node metastasis		
N0	89	40.83
N1–2	129	59.17
Liver metastasis		
M0	106	48.62
M1	112	51.38
TNM stage		
I + II	75	34.40
III + IV	143	65.60

### Quantitative real-time PCR

Total RNA was extracted from 56 pairs of fresh tissues using Trizol reagent (Life Technologies), according to the manufacturer’s instructions, and the RNA concentration was determined using an Ultrospec^®^ 3300 Pro (GE Health Care, Little Chalfont, Buckinghamshire, UK). Reverse transcription was performed using 4000 ng of total RNA in a 20 μL reaction volume using the EasyScript First-Strand cDNA Synthesis SuperMix Kit (Life Technologies). The mRNA expression of CD147 and MMP-11 was examined by real-time PCR using Power SYBR^®^ Green PCR Master Mix (Life Technologies) with gene-specific primers and the ABI 7500 Real-time PCR Detection System. The primer sequences were as follows: CD147: 5′-GGCTGTGAAGTCGTCAGAACAC-3′ (sense) and 5′-ACCTGCTCTCGGAGCCGTTCA-3′ (antisense); MMP-11: 5′-GAGAAGACGGACCTCACCTACA-3′ (sense) and 5′-CTCAGTAAAGGTGAGTGGCGTC-3′ (antisense); beta-actin: 5′-TTAGTTGCGTTACACCCT TTC-3′ (sense) and 5′-ACCTTCACCAGTTCCAGTTT-3′ (antisense). The thermal cycling conditions involved predenaturation at 95 °C for 10 min, followed by 40 cycles of denaturation at 95 °C for 15 s, annealing at 60 °C for 1 min, and extension at 72 °C for 30 s. All reactions were performed in triplicate. The mRNA expression was measured using threshold cycle values (C_t_). To normalize this value, a ΔC_t_ value was determined as the difference between the C_t_ value for each gene and the C_t_ value for β-actin. Then, a ΔΔC_t_ value was determined as the difference between the ΔC_t_ value for each individual sample and the average ΔC_t_ value for this gene, obtained from the control samples. The results were presented as fold changes, calculated using the 2^−ΔΔCt^ method (Livak and Schmittgen 2001). The comparative C_t_ method, normalizing C_t_ values to β-actin, was used to calculate the relative expression level of CD147 and MMP-11.

### Colorectal cancer tissue microarray (TMA) construction

All specimens were stained with hematoxylin and erosin, and matched representative cancerous and adjacent normal mucosa from paraffin blocks were isolated from 218 colorectal cancer patients. For each patient, three cylinders (the diameter is 1.0 mm) of representative cancerous paraffin blocks and two cylinders (the diameter is 1.0 mm) of normal mucosa paraffin blocks were obtained. To obtain a broad coverage of the tumor tissue, the three regions were preferentially chosen from the tumor center and the invasive margins. Then, the representative cylinders were arrayed into a receptor paraffin block by an automated tissue-arraying instrument (ALPHELYS) and reheated for 30 min at 37 °C. The 4 μm sections were then cut from the resulting TMA block.

### Immunohistochemistry assay

TMA block sections (4 μm thick) were baked at 70 °C, dewaxed with xylene, and rehydrated with graded alcohol washes. Antigen retrieval was performed in a pressure cooker, followed by the treatment with 3 % hydrogen peroxide for 15 min to block endogenous peroxidase activity. Thereafter, the sections were incubated at 4 °C overnight with anti-CD147 (ab78106, abcam) or anti-MMP-11(ab52904, abcam). The stained specimens were exposed to the 3,3-diam-inobenzidine (DAB) Kit (Zhong shan Biotechnology Inc., Beijing, China) and counterstained with hematoxylin. For the negative controls, primary antibodies were replaced with PBS.

### Immunofluorescence assay

Double-fluorescence staining of CD147 and MMP-11 were conducted on formalin-fixed, paraffin-embedded tissue sections from 20 CRC patients. The slides were baked at 70 °C, dewaxed with xylene, and rehydrated with graded alcohol washes. Antigen retrieval was performed in a pressure cooker, followed by the treatment with 3 % hydrogen peroxide for 15 min to block endogenous peroxidase activity. Thereafter, the sections were incubated at 4 °C overnight with anti-CD147 (ab78106, abcam) or anti-MMP-11(ab52904, abcam). Then the CD147 and MMP-11 primary antibodies were detected by secondary antibodies as follows: Goat anti-mouse IgG (Zhong shan Biotechnology Inc., Beijing, China, ZF-0312, green) and Goat anti-rabbit IgG (Zhong shan Biotechnology Inc., Beijing, China, ZF-0316, red). After washing for two times, nuclei were stained with 4′,6′- diamidino-2-phenylindole (DAPI, Zhong shan Biotechnology Inc., Beijing, China, ZLI-9557) at room temperature for 10 min, and stored at 4 °C. The slides were examined using a laser scanning confocal microscope (Zeiss LSM510). Images were collected and processed using the Zeiss AIM software and sized in Adobe Photoshop CS6.

### Evaluation immunohistochemical of staining

The CD147 and MMP-11 staining were microscopically examined and scored by two independent pathologists who were blind to the clinical data pertaining to the patients. For CD147 and MMP-11 immunohistochemical staining assessment, immunoreactivity score (IRS) was assessed which evaluated both the percentage of positive cells and the staining intensity. The percentage of positive cells was scored as 0 (negative), 1 (<25 %), 2 (25–75 %), and 3 (>75 %); staining intensity was graded as 0 (colorless), 1 (pallide-flavens), 2 (yellow), and 3 (brown). The above two scores were multiplied, and “negative” and “positive” expression were defined according to IRS values of 0 and >0, respectively.

### Statistical analysis

Statistical analysis was carried out using SPSS software package version 16.0 (SPSS Inc., Chicago, IL, USA). The differential expression of CD147 and MMP-11 mRNA between colorectal cancer tissues and adjacent normal mucosa was compared using a nonparametric test. Two-tailed *χ*^2^ test was used to evaluate the expression difference between colorectal cancer tissue and normal mucosa, as well as the relationship between the clinicopathological features and CD147 or MMP-11 expression. Correlation between CD147 and MMP-11was evaluated using the Spearman rank test. The survival curves were estimated by Kaplan–Meier analysis, and *P* values were calculated by log rank test. The effect of different factors on patient survival was performed by multivariate analysis with the Cox proportional hazards regression model. *P* < 0.05 was considered significant.

## Results

### mRNA expression of CD147 and MMP-11 in fresh, paired CRC tissues

**Fig. 1:**
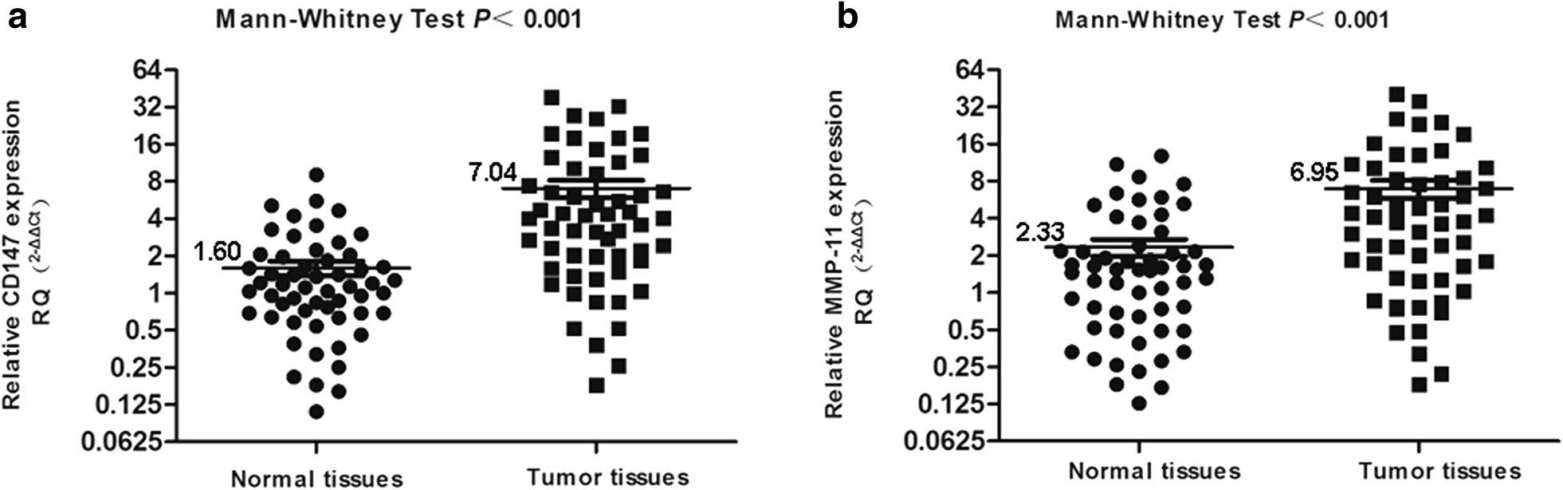
Real-time PCR evaluation of CD147 and MMP-11 mRNA expression in 14 pairs of CRC tissues and matched normal mucosa. **a** mRNA expression of CD147 in tumor tissues was higher than that in normal tissues (*P* = 0.002). **b** mRNA expression of MMP-11 in tumor tissues was higher than that in normal tissues, too (*P* = 0.043). The relative quantification (RQ) was calculated using the 2^−ΔΔCt^method

Both CD147 and MMP-11 mRNA expression levels in 56 pairs of primary CRC and matched normal mucosa were examined using real-time PCR. Results showed that the mRNA levels of both CD147 and MMP-11 were much higher in tumor tissues than in normal tissues (*P* < 0.001, both) (Fig. 1).

### CD147 and MMP-11 expression in CRC tissues by IHC and immunofluorescence double staining

**Fig. 2:**
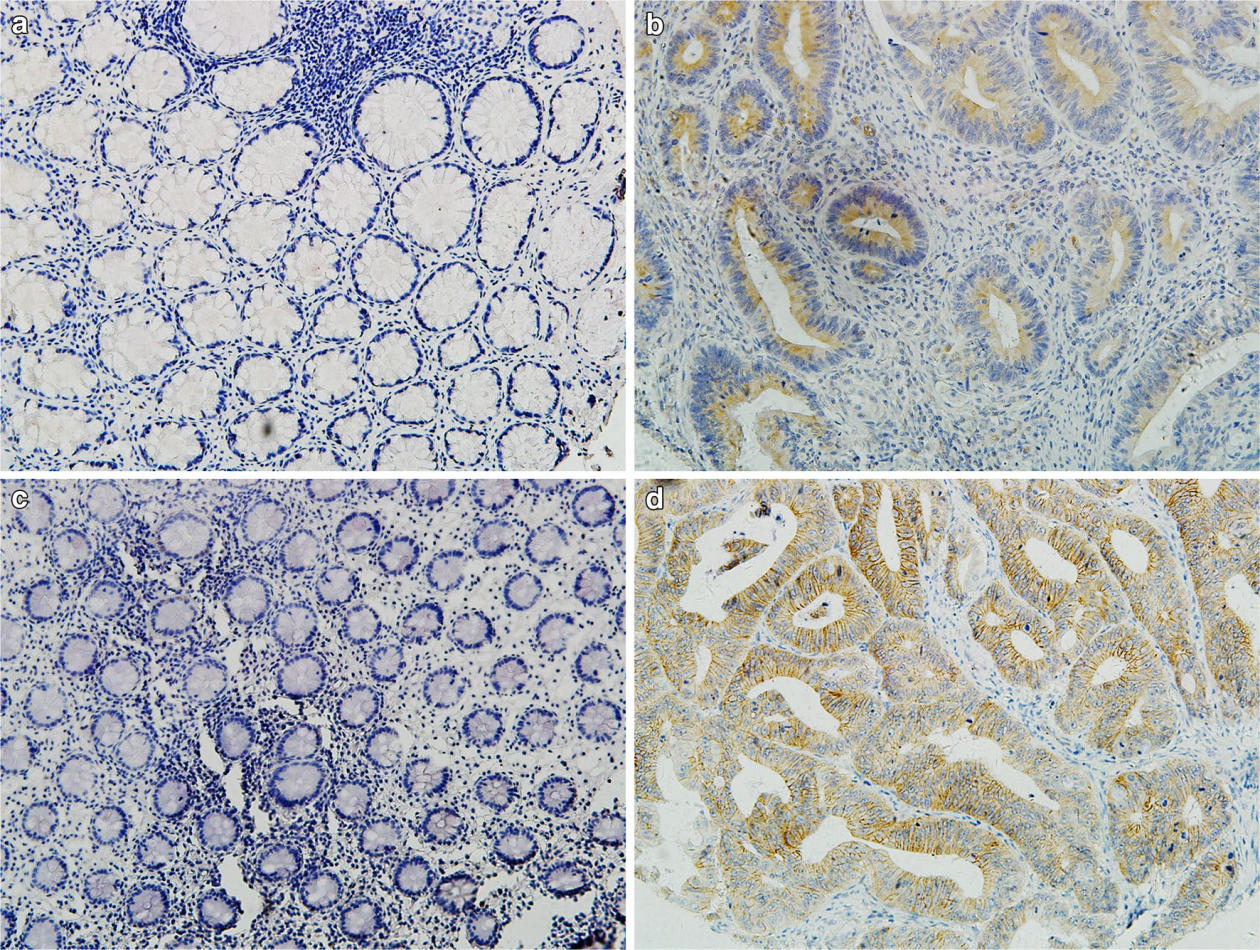
Representative immunohistochemical staining of CD147 and MMP-11 in CRC tissue and matched normal mucosa. **a**, **b** show CD147 staining in normal mucosa and CRC tissue, respectively (magnification ×100). **c**, **d** show MMP-11 staining in normal mucosa and CRC tissue, respectively (magnification ×100). As is shown, CD147 protein is predominantly localized on cell membrane and in the cytoplasm, while MMP-11 protein was of cytoplasmic staining

**Fig. 3:**
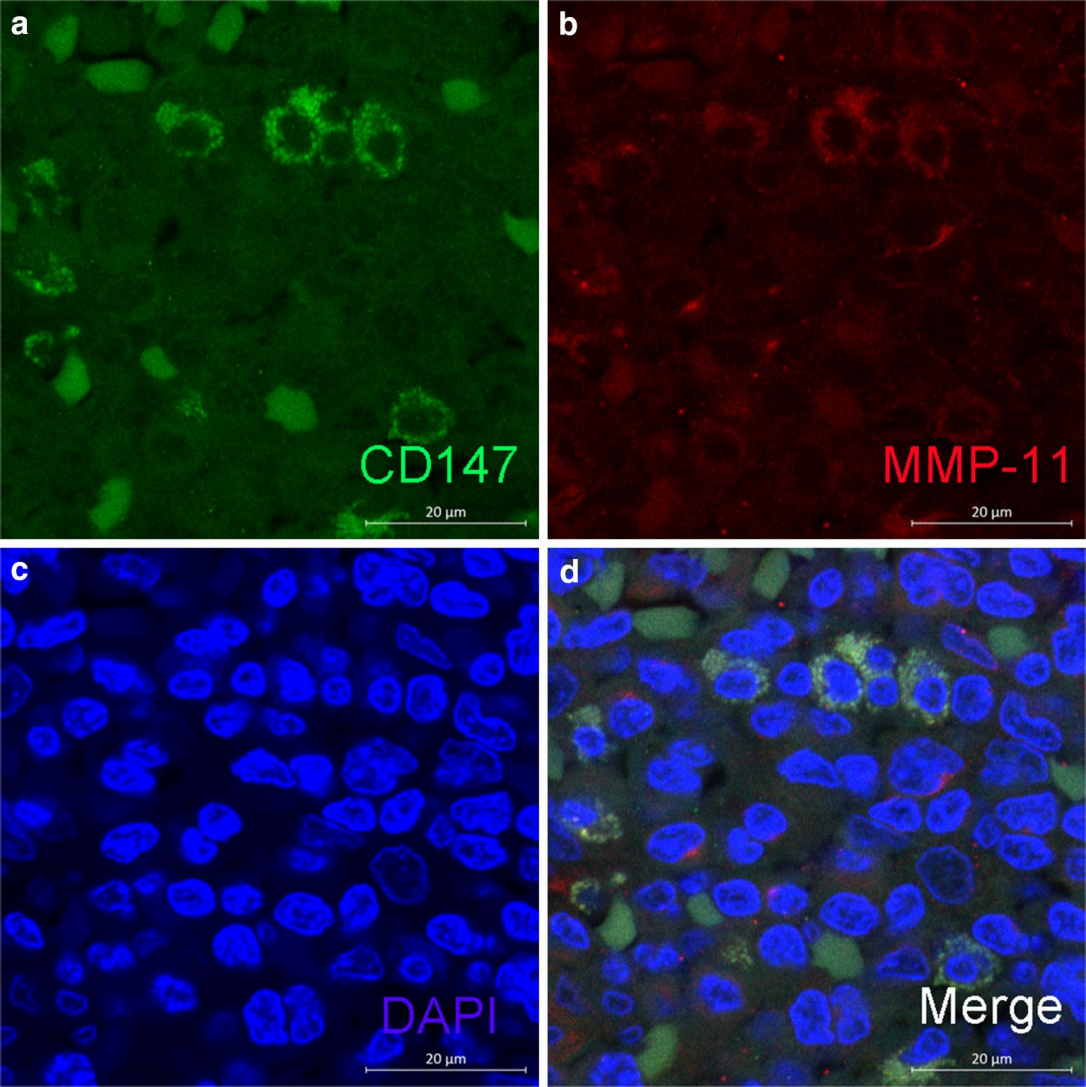
Representative photomicrographs of double fluorescent staining of CD147 with MMP-11. Both **a** CD147 (*green*) and **b** MMP-11 (*red*) showed a predominant membranous and cytoplasmic localization. **c** DAPI (*blue*) was stained to identify nuclei. **d** Co-localization of CD147 and MMP-11 (*yellow*) is apparent. *Scale bar* 20 µm

Immunohistochemical staining showed a membranous and cytoplasmic staining of both CD147 and MMP-11 of tumor cells (Fig. 2). CD147 expression in normal mucosa was significantly lower than that in colorectal cancer (9.17 vs. 59.63 %, *P* < 0.001). Similarly, there was also a significantly higher expression of MMP-11 in colorectal cancer tissue than in normal mucosa (77.06 vs. 33.94 %, *P* < 0.001) (see Table 2). Spearman’s rank correlation coefficient test showed a positive correlation between CD147 and MMP-11 expression in CRC tissues (*r*_*s*_ = 0.152, *P* = 0.025). Immunofluorescence double staining assay confirmed the intracellular localization of CD147 and MMP-11 shown in immunohistochemical staining, and evident co-localization can be observed between these two proteins (Fig. 3).

**Table 2:** Expression of CD147 and MMP-11 in carcinoma tissue and matched normal mucosa of CRC patients

Groups	CD147 expression			MMP-11 expression		
	Positive (%)	Negative (%)	*P* value	Positive (%)	Negative (%)	*P* value
Carcinoma tissue	135 (61.93)	83 (38.07)	<0.001	168 (77.06)	50 (22.94)	<0.001
Normal mucosa	21 (9.63)	197 (90.37)		74 (33.94)	144 (66.06)

### Association of immunohistochemical expression of CD147 and MMP-11 with clinicopathological features

Based on staining evaluation and the statistical analysis, we further investigated the correlation between CD147/MMP-11 expression and the clinicopathological features as shown in Table 3. CD147 expression was markedly higher in patients with lymph node metastasis (*P* = 0.021), distant metastasis (*P* < 0.001), and advanced TNM stage (*P* = 0.006). Interestingly, MMP-11 expression was also significantly correlated with the above three parameters, with *P* = 0.031, 0.013, 0.049, respectively. However, both CD147 and MMP-11 expression was not correlated with patient’s gender, age, differentiation, tumor size, and depth of invasion. Detailed results are shown in Table 3.

**Table 3:** Association of CD147 and MMP-11 expression with clinicopathological features

Clinicopathological features	CD147 expression			MMP-11 expression		
	Positive (%)	Negative (%)	*P* value	Positive (%)	Negative (%)	*P* value
Gender						
Male	83 (64.34)	46 (35.66)	0.377	99 (76.74)	30 (23.26)	0.892
Female	52 (58.43)	37 (41.57)		69 (77.53)	20 (22.47)	
Age (years)						
<60	57 (57.58)	42 (42.42)	0.228	77 (77.78)	22 (22.22)	0.819
≥60	78 (65.53)	41 (34.45)		91 (76.47)	28 (23.53)	
Differentiation						
Poor	26 (66.67)	13 (33.33)	0.501	32 (82.05)	7 (17.95)	0.414
Moderate-well	109 (60.89)	70 (39.11)		136 (75.98)	43 (24.02)	
Size						
<4 cm	62 (59.62)	42 (40.38)	0.502	81 (77.88)	23 (22.12)	0.783
≥4 cm	73 (64.04)	41 (35.96)		87 (76.32)	27 (23.68)	
Lymphovascular invasion						
Absent	93 (62.42)	56 (37.58)	0.827	110 (73.83)	39 (26.17)	0.095
Present	42 (60.87)	27 (39.13)		58 (84.06)	11 (15.94)	
Depth of invasion						
T1/T2	16 (48.48)	17 (51.52)	0.084	22 (66.67)	11 (33.33)	0.123
T3/T4	119 (64.32)	66 (35.68)		146 (78.92)	39 (21.08)	
Lymph node metastasis						
N0	47 (52.81)	42 (47.19)	0.021	62 (69.66)	27 (30.34)	0.031
N1–2	88 (68.22)	41 (31.78)		106 (82.18)	23 (17.83)	
Distant metastasis						
M0	53 (63.86)	30 (36.14)	<0.001	74 (69.81)	32 (30.19)	0.013
M1	53 (39.26)	82 (60.74)		94 (83.93)	18 (16.07)	
TNM stage						
I + II	37 (49.33)	38 (50.67)	0.006	52 (69.33)	23 (30.67)	0.049
III + IV	98 (68.53)	45 (31.47)		116 (81.11)	27 (18.89)	

### Prognostic implication of CD147 and MMP-11 in colorectal cancer

**Fig. 4:**
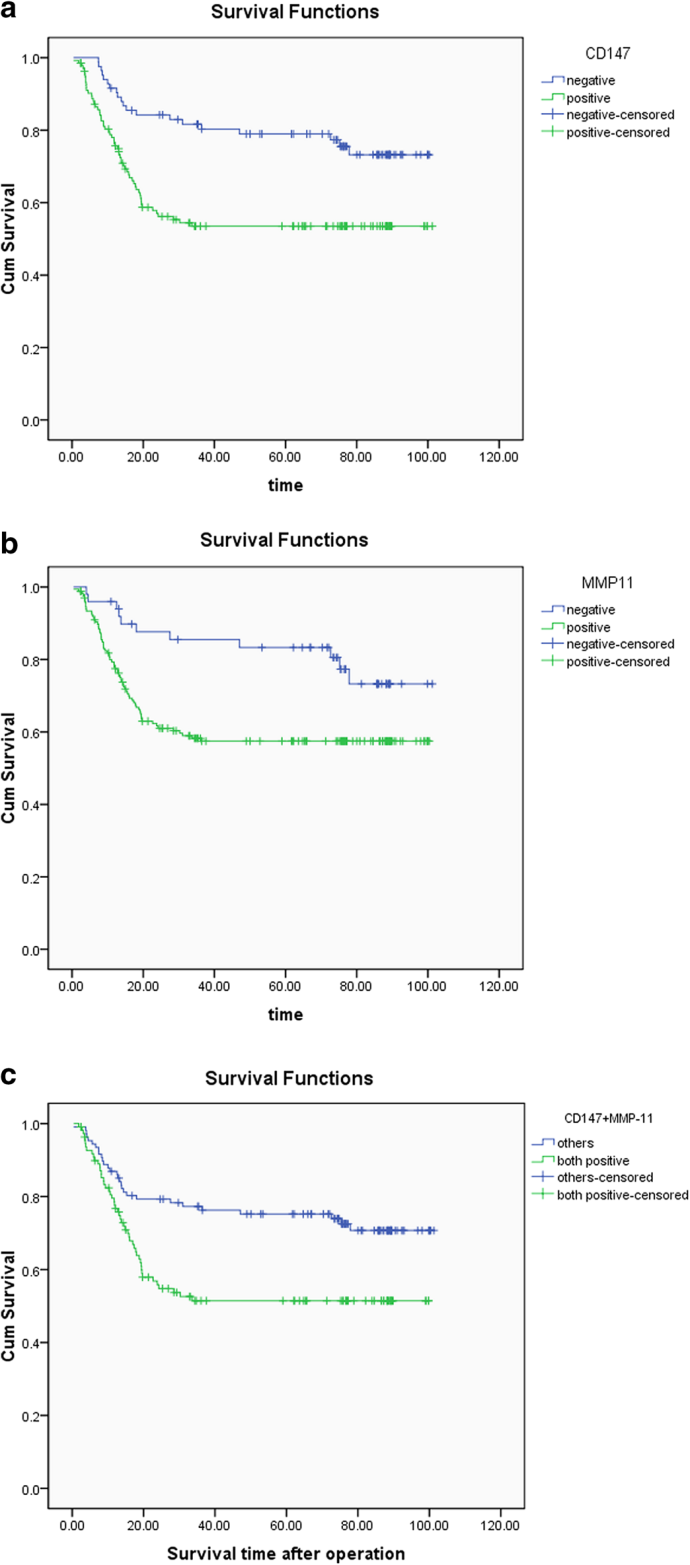
Kaplan–Meier overall survival analysis of colorectal cancer patients. Survival analysis was performed according to the expression status of **a** CD147 (*P* = 0.001), **b** MMP-11 (*P* = 0.009), and **c** CD147 combined with MMP-11 (*P* = 0.002), respectively

The follow-up time of all patients was from February 2006 to July 2014. The overall median survival time was 49.35 months. During the follow-up period, a total of 79 patients died of colorectal cancer. The survival curves were estimated by Kaplan–Meier analysis and then compared using the log-rank test. Results showed that patients with CD147 or MMP-11 expression had a poorer prognosis (*P* = 0.001 and 0.009, respectively) (Fig. 4). To test the prognostic value of combined expression status of CD147 and MMP-11, we classified all patients into two groups, CD147+/MMP-11+ group and all other expression statuses as the other group. Kaplan–Meier analysis showed that patients with CD147+/MMP-11+ had a significantly poor survival time than those with other expression statuses (*P* = 0.002). Then, to test whether CD147 and MMP-11 expression were independent prognostic factors for CRC patients, we performed a multivariate survival analysis, in which those parameters associated with overall survival in the univariate survival analysis were included. In univariate analysis, CD147 expression (*P* = 0.001), MMP-11 expression (*P* = 0.011), differentiation (*P* < 0.001), lymphovascular invasion (*P* < 0.001), and tumor staging (*P* < 0.001) were associated with overall survival. In multivariate analysis lymphovascular invasion, CD147, MMP11 and tumor staging were independent prognostic factors related to overall survival (*P* = 0.044, 0.009, 0.028, <0.001, respectively). Detailed data are shown in Table 4.

**Table 4:** Cox proportional hazard regression model analysis

Variables	Univariate Cox’s regression analysis			Multivariate Cox’s regression analysis		
	Relative risk	95 % CI	*P* value	Relative risk	95 % CI	*P* value
Differentiation						
Moderate-well versus poor	0.397	0.244–0.646	<0.001			
Lymphovascular invasion						
Present versus absent	3.606	2.310–5.627	<0.001	1.611	1.013–2.561	0.044
CD147 expression						
Positive versus negative	2.364	1.422–3.931	0.001	2.023	1.193–3.430	0.009
MMP-11 expression						
Positive versus negative	2.281	1.206–4.316	0.011	2.094	1.083–4.047	0.028
T stage						
T1 + T2 versus T3 + T4	4.432	1.619–12.132	0.004			
N stage						
N0 versus N1 + N2 + N3	6.616	3.486–12.556	<0.001			
Metastasis						
No versus yes	32.746	12.946–82.860	<0.001	25.970	10.001–67.442	<0.001

## Discussion

Local invasion and distant metastasis are suggested to be the key reasons for poor prognosis and cancer related death in tumor patients. Both these two proteins investigated in this study are closely related with tumor invasion and metastasis. In this study we found that there was an increased expression of CD147 and MMP-11 in colorectal cancer compared with paired normal mucosa, suggesting that CD147 and MMP-11 might play an oncogenic role in colorectal cancer.

It has been well documented that CD147 induces the expression of several MMPs, such as MMP-1, MMP-2, MMP-9 [12], which are associated with tumor migration and invasion. The mechanisms by which CD147 up-regulates MMPs family appear to depend on its stimulation of the tumor-associated fibroblasts. The expression association between CD147 and MMP-2, MMP-9 has been well established in a series of human malignant tumors, such as gallbladder carcinoma, thyroid carcinoma, breast cancer, gastric cancer and colorectal cancer. While up to now, reports on the relationship between CD147 and MMP-11 are limited. Jia et al. reported that in murine hepatocarcinoma Hca-F cells, depletion or deglycosylation of CD147 down-regulated MMP-11 expression [13]. In another study, knockdown of CD147 reduced the secretion of MMP-11 in nasopharyngeal carcinoma [14]. In our study, we explored the relationship between CD147 and MMP-11 expression in colorectal cancer, and found a positive correlation between these two proteins, and immunofluorescence double staining showed co-localization of these two proteins, which has provided new evidence for the regulatory interaction between CD147 and MMP-11.

Recent studies have established a role of oncogenic and tumor promoter for CD147, and its expression has been proved to be related with clinicopathological characteristics in a variety of cancer types. Zhong et al. [15] found that CD147 expression was correlated with higher incidence of lymph node metastasis and lower differentiation in stage T1 pulmonary adenocarcinoma; Zhao et al. [16] found that CD147 expression was correlated with tumor invasion and metastasis in triple-negative breast cancer; in human astrocytomas and meningiomas, overexpression of CD147 was found in high-grade tumors, and it was positively correlated with WHO grades [17]. What’s more, CD147 expression has always been associated with a poor survival of cancer patients [18, 19]. As for colorectal carcinoma, previous studies have got similar results. Zheng et al. [20] found that CD147 protein was up-regulated in colorectal cancer, without the alteration of its glycosylation or mRNA level. Stenzinger showed in their study that CD147 expression was associated with clinical TNM stage of CRC [21]. A recent study showed that CD147 was an independent prognostic factor for disease-free survival of CRC patients [22]. In our study, we have found that CD147 expression was correlated with lymph node metastasis, distant metastasis, as well as TNM stage for CRC; What’s more, it is an independent prognostic factor for CRC patients. Our results have highlighted the important role of CD147 in the metastasis of CRC and its prognostic value in patients, both of which suggesting its clinical significance. Recently, some novel roles of CD147 in cancer have also been found. Xu et al. [23] uncovered a role of CD147 in transforming fibroblasts to cancer-associated fibroblasts, which in turn induced epithelial-to-mesenchymal (EMT) transition of breast cancer cells. These findings support a novel role of CD147 in regulating the interaction between cancer and stroma. Hibino et al. [24] found that the calcium-binding proteins S100A9 may serve as a novel ligand for CD147 to promote melanoma metastasis. ERK1/2 signaling pathway was found to be involved in CD147-mediated proliferation and invasion of gastric cancer cell line SGC7901 [25]. In another study, hypoxic microenvironment was postulated to be a major initiator of the overexpression of CD147 [26]. In addition, it has been reported that CD147 might be involved in drug resistance in different cancer types, via different mechanisms [22, 27]. The above discoveries suggested that CD147 can be involved in several malignant transformation processes other than inducing MMPs, and it may exert its function as a member in networks, instead of a dominant sector, maybe that’s why discrepancies exist in different studies.

As a member of the MMPs family, MMP-11 has long been postulated to play an important role in tumorigenesis, invasion, metastasis and poor clinical outcome in malignant tumors. While owing to its complex biological property, the role of MMP-11 has not been that established as other MMPs, such as MMP-2 and MMP-9. The mechanism by which MMP-11 participates in tumor progression is also unique. On the one hand, it belongs to the MMPs family, so it possesses some common properties of the family; on the other hand, it is devoid of enzymatic activity against the matrix components which is thought to be the most prominent feature of the family, so it may not have that much power in paving the path for tumor cells during their migration and invasion into the healthy tissue effectively just like other MMPs. In a study conducted by Boulay et al. [28], a new and unexpected role for MMP was revealed, that is, tumorigenesis induced by MMP-11 does not result from increased neo-angiogenesis or cancer cell proliferation, but from decreased cancer cell death through apoptosis and necrosis, indicating that the cellular function of MMP-11 is to favor cancer cell survival in the stromal environment. Similarly, MMP-11may also play a role in lobular carcinogenesis through increasing resistance to anoikis [9]. Recently a study reported the relationship between MMP-11 and inflammation. MMP-11 expression by mononuclear inflammatory cells (MICs) coupled with the expression of pro-inflammatory proteins support tumor escape and invasion, hence promoting metastasis, so they claim that the expression of MMP-11 may constitute a useful biological marker for pro-metastatic MICs [29].

Interestingly, there might also be a dual role of MMP-11 in cancers. It plays an oncogenic role in cervical cancer [30] and gastric cancer [31]. It can also serve as a predictor of prognosis, overexpression of MMP-11 was associated with poor prognosis in breast ductal carcinoma [8] and advanced gastric cancer [10]. In our study, it is significantly related with survival of CRC patients and can also serve as an independent prognostic factor in multivariate analysis; what’s more, MMP-11 was associated with lymph node metastasis and distant metastasis, and also with tumor’s TNM stage, which was similar with results in non-small cell lung cancer and breast cancer [32, 33]. However, Brasse et al. [34] found a paradoxical role of MMP-11 during the hematogenous metastatic process in mice. In their research, MMP-11 favors the onset and growth of lung metastasis but limiting lung foci number, and inhibiting the cancer cell dissemination to other organs. In another study, there was an inverse relationship between MMP-11 expression and predictors of poor prognosis of papillary thyroid carcinoma [35]. All the reported results indicated a multidimensional role of MMP 11 in cancer. If this is also the situation with other MMPs, although it’s just a bold presume without enough evidence up to date, maybe the dismal curative effect of the anti-MMPs new drugs can be explained. Studies with more cases and more functional investigation are necessary.

## Conclusions

Our study has demonstrated that CD147 and MMP-11 are overexpressed in colorectal cancer, and there’s a positive correlation and co-localization between these two proteins. Both CD147 and MMP-11 might act as tumor promoters during the progression of CRC patients. What’s more, the two proteins are associated with patients’ survival. These indicated that CD147 and MMP-11 might become potential prognostic markers as well as therapeutic targets for CRC patients.

## Abbreviations

CRC: colorectal cancer
MMPs: matrix metalloproteinases
ECM: extracellular matrix
EMMPRIN: extracellular matrix metalloproteinase inducer
TMA: tissue microarray
